# Supplementary material for: Polyketide synthase gene clusters in the ericoid mycorrhizal fungus Oidiodendron maius: genome-wide prediction, expression profiling, and functional characterization of knockout mutants
Source: Front Plant Sci. 2026 Sep 16;17:1924430. doi: 10.3389/fpls.2026.1924430 (PMC13623903; doi:10.3389/fpls.2026.1924430)
Supplement: Supplementary file 1 [file SupplementaryFile1.zip › Supplementary Materials/Supplementary Folder 1.zip!/Oidma1_AssemblyScaffolds_Repeatmasked/knownclusterblast/cluster9/jgi.p_Oidma1_100477_mibig_hits.html]

| MIBiG Protein | Description | MIBiG Cluster | MiBiG Product | % ID | % Coverage | BLAST Score | E-value |
| --- | --- | --- | --- | --- | --- | --- | --- |
| BAI63273.1 | putative\_kynurenine\_aminotransferase | BGC0000434 | SW-163\_biosynthetic\_gene\_cluster | 26 | 48.4107579462 | 77 | 5e-14 |
| CAJ34363.1 | kynurenine\_aminotransferase | BGC0000445 | Thiocoraline\_biosynthetic\_gene\_cluster | 22 | 76.0391198044 | 64 | 4.4e-10 |
| ANW12120.1 | aminotransferase | BGC0001374 | Thienodolin\_biosynthetic\_gene\_cluster | 28 | 52.3227383863 | 61 | 3.7e-09 |
